# Lipid Nanoparticles Decorated with TNF-Related Aptosis-Inducing Ligand (TRAIL) Are More Cytotoxic than Soluble Recombinant TRAIL in Sarcoma

**DOI:** 10.3390/ijms19051449

**Published:** 2018-05-13

**Authors:** Ana Gallego-Lleyda, Diego De Miguel, Alberto Anel, Luis Martinez-Lostao

**Affiliations:** 1 Departamento de Bioquímica, Biología Molecular y Celular, Universidad de Zaragoza, 50009 Zaragoza, Spain; annaiss89@hotmail.com (A.G.-L.); diego_demiguel@hotmail.com (D.D.M.); anel@unizar.es (A.A.); 2 Instituto de Investigación Sanitaria de Aragón (ISS), 50009 Zaragoza, Spain; 3 Cell Death, Cancer and Inflammation, University College of London, London WC1E 6BT, UK; 4 Servicio de Inmunología, Hospital Clínico Universitario Lozano Blesa, 50009 Zaragoza, Spain; 5 Departamento de Microbiología, Medicina Preventiva y Salud Pública, Universidad de Zaragoza, 50009 Zaragoza, Spain; 6 Instituto de Nanociencia de Aragón, 50009 Zaragoza, Spain

**Keywords:** sarcoma, TRAIL, flavopiridol, immunotherapy, lipid nanoparticles

## Abstract

Sarcomas are rare and heterogeneous cancers classically associated with a poor outcome. Sarcomas are 1% of the cancer but recent estimations indicate that sarcomas account for 2% of the estimated cancer-related deaths. Traditional treatment with surgery, radiotherapy, and chemotherapy has improved the outcome for some types of sarcomas. However, novel therapeutic strategies to treat sarcomas are necessary. TNF-related apoptosis-inducing ligand (TRAIL) is a death ligand initially described as capable of inducing apoptosis on tumor cell while sparing normal cells. Only few clinical trials have used TRAIL-based treatments in sarcoma, but they show only low or moderate efficacy of TRAIL. Consequently, novel TRAIL formulations with an improved TRAIL bioactivity are necessary. Our group has developed a novel TRAIL formulation based on tethering this death ligand on a lipid nanoparticle surface (LUV-TRAIL) resembling the physiological secretion of TRAIL as a trasmembrane protein inserted into the membrane of exosomes. We have already demonstrated that LUV-TRAIL shows an improved cytotoxic activity when compared to soluble recombinant TRAIL both in hematological malignancies and epithelial-derived cancers. In the present study, we have tested LUV-TRAIL in several human sarcoma tumor cell lines with different sensitivity to soluble recombinant TRAIL, finding that LUV-TRAIL was more efficient than soluble recombinant TRAIL. Moreover, combined treatment of LUV-TRAIL with distinct drugs proved to be especially effective, sensitizing even more resistant cell lines to TRAIL.

## 1. Introduction

Sarcomas include a heterogeneous group of complex cancers which derive from mesenchymal transformed cells. Sarcomas are rare, making up for about 1% of all cancers [1]. It is well known that prognosis of sarcomas is poor, with a 5-year survival rate of less than 15% when metastasis happens [2]. Over the last years, a great effort for improving treatment outcome has been made, but the complexity of sarcomas makes that advancements in developing new therapies are extremely slow [3]. Therefore, there is an urgent need to develop and test novel therapeutic strategies in order to improve prognosis of sarcoma patients [4]. Among the novel anti-tumor agents used, apoptosis ligand 2/TNF-related apoptosis-inducing ligand (Apo2L/TRAIL) has been tested both in pre-clinical studies and clinical trials in some types of sarcoma [5].

Apoptosis ligand 2/TNF-related apoptosis-inducing ligand (Apo2L/TRAIL) is a TNF family member described as capable of inducing apoptosis in a wide variety of transformed cells while sparing normal cells [6,7]. This fact made TRAIL to be considered as a promising anti-tumor agent. In fact, TRAIL-based therapeutic strategies were developed and used in several phase I/II clinical trials on a wide variety of human cancers [8,9,10]. However, although TRAIL-based therapies were proven safe, about 50% of human cancers were intrinsically resistant to TRAIL since the clinical studies using TRAIL showed limited therapeutic activity of this death ligand in different types of cancer [9,11,12] including sarcoma [5]. Therefore, novel formulations of TRAIL with improved bioactivity and combination of TRAIL with sensitizing agents could be plausible strategies to overcome TRAIL resistance [13,14,15,16].

Our group generated artificial lipid nanoparticles containing membrane-bound TRAIL (LUV-TRAIL), resembling the natural TRAIL-containing exosomes physiologically released by activated human T cells upon re-stimulation [17,18]. We demonstrated that LUV-TRAIL are more effective than soluble recombinant TRAIL (sTRAIL), inducing apoptosis both in vitro and in vivo in hematological malignancies [19,20,21] as well as in epithelial-derived cancers [22,23,24].

In the present work, we have tested LUV-TRAIL in several human sarcoma cell lines of distinct origin with different sensitivity to sTRAIL. LUV-TRAIL were more efficient inducing apoptosis in all sarcoma cells tested than sTRAIL. However, although LUV-TRAIL showed a greater pro-apoptotic potential compared to sTRAIL, some sarcoma cell lines still remained fairly resistant to LUV-TRAIL. In this line, a wide range of sensitizing strategies have been described to overcome TRAIL-resistance in tumor cells. Here, we have tested the therapeutic combination of LUV-TRAIL with several drugs previously described as sensitizing agents [23,24,25,26,27,28,29,30], in order to improve the pro-apoptotic ability of LUV-TRAIL. Our results showed that, among all the combinations tested, flavopiridol induced the strongest sensitizing effect in all sarcoma cell lines tested, by inducing a down-regulation of the anti-apoptotic protein FLIP.

In summary, LUV-TRAIL showed an improved cytotoxicity against sarcoma cells, overcoming the intrinsic resistance of these cells to sTRAIL. This was further potentiated when they were combined with sensitizing agents such as flavopiridol (FVP), opening the door to future clinical applications as anti-tumor therapy in sarcoma.

## 2. Results

### 2.1. LUV-TRAIL Showed an Enhanced In Vitro Cytotoxic Activity Compared to Soluble Recombinant TRAIL in Human Sarcoma Cells

**Figure 1:**
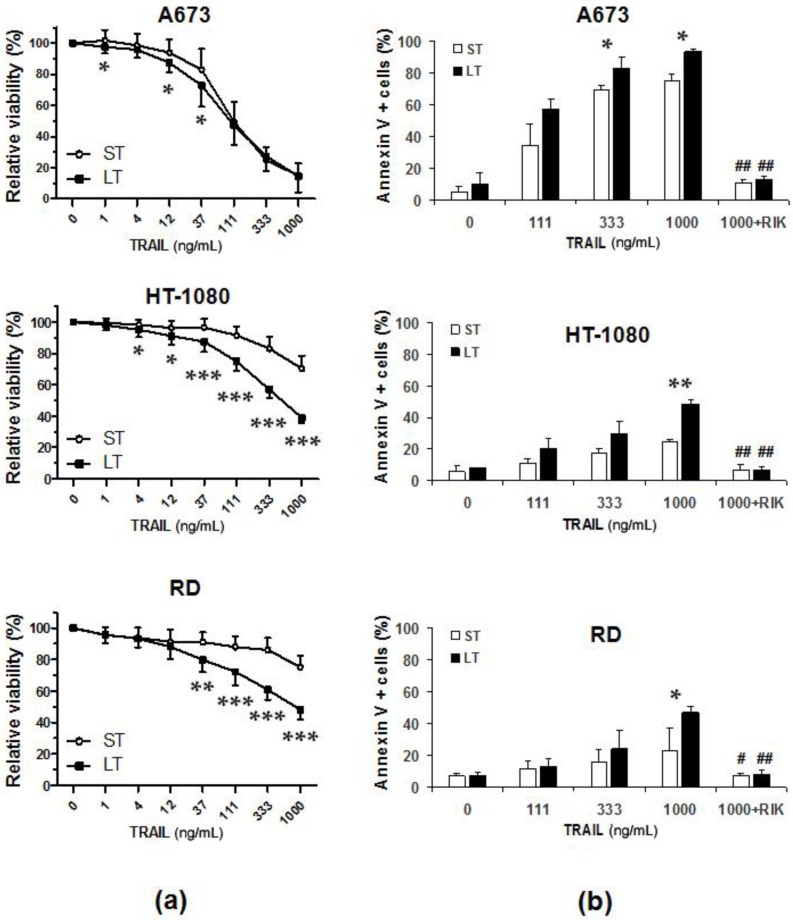
(**a**) Analysis of cell viability after treatment with LUV-TRAIL on human sarcoma cell lines. Dose-response assays using the indicated doses of sTRAIL (ST) and LUV-TRAIL (LT) were performed on A673 cells, HT-1080 cells, and RD cells. Cells were treated with ST or LT for 24 h. Then, cell viability was measured by the MTT assay method. Graphs show the mean ± SD of at least three independent experiments. * *p* < 0.05, ** *p* < 0.01, *** *p* < 0.001; (**b**) Cytotoxicity assays on human sarcoma cell lines. Cells were treated with indicated doses of sTRAIL (ST) or LUV-TRAIL (LT) for 24 h and annexin V positive cells were quantified by flow cytometry. When cells were treated with 1000 ng/mL, they were previously pre-incubated in presence or absence of the anti-TRAIL blocking mAb, RIK2 (500 ng/mL). Graphics show the percentage of annexin-V positive cells analyzed expressed as the mean ± SD of at least three experiments. * *p* < 0.05. (ST versus LT). # *p* < 0.05, ## *p* < 0.01 (ST versus ST + RIK2 and, LT versus LT + RIK2). TRAIL, TNF-related apoptosis-inducing ligand; LUV-TRAIL, TRAIL on a lipid nanoparticle surface; sTRAIL, soluble recombinant TRAIL.

First, dose-response assays were carried out to compare the in vitro bioactivity of sTRAIL and LUV-TRAIL, and cell viability was analyzed by the MTT assay (Figure 1a). Whereas A673 cells showed a great sensitivity both to sTRAIL and LUV-TRAIL, HT-1080 and RD cell lines showed a moderate sensitivity to sTRAIL However, LUV-TRAIL was capable of inducing a significant decrease of cell viability both in HT-1080 and RD cells in comparison with sTRAIL. To assess whether the decrease in cell viability observed after treatment with both forms of TRAIL (sTRAIL and LUV-TRAIL) was due to the onset of cell death, annexin-V staining was performed (Figure 1b). In all sarcoma cell lines tested, LUV-TRAIL induced a remarkable increase of cell death at higher dose used (1000 ng/mL) when compared to sTRAIL. Importantly, the cell death observed in all sarcoma cell lines was specifically attributable to TRAIL-receptor activation by TRAIL, as cell death was completely inhibited when cells were pre-incubated with the TRAIL neutralizing antibody RIK2 before treatment with sTRAIL and LUV-TRAIL (Figure 1b). In this line, LUVs alone (without TRAIL anchoring on their surface), did not exert any cytotoxic effect in any sarcoma cell lines tested (see black bars on control points in Figure 1b).

### 2.2. LUV-TRAIL Activated the Caspase Cascade More Efficiently than sTRAIL in Human Sarcoma Cells

**Figure 2:**
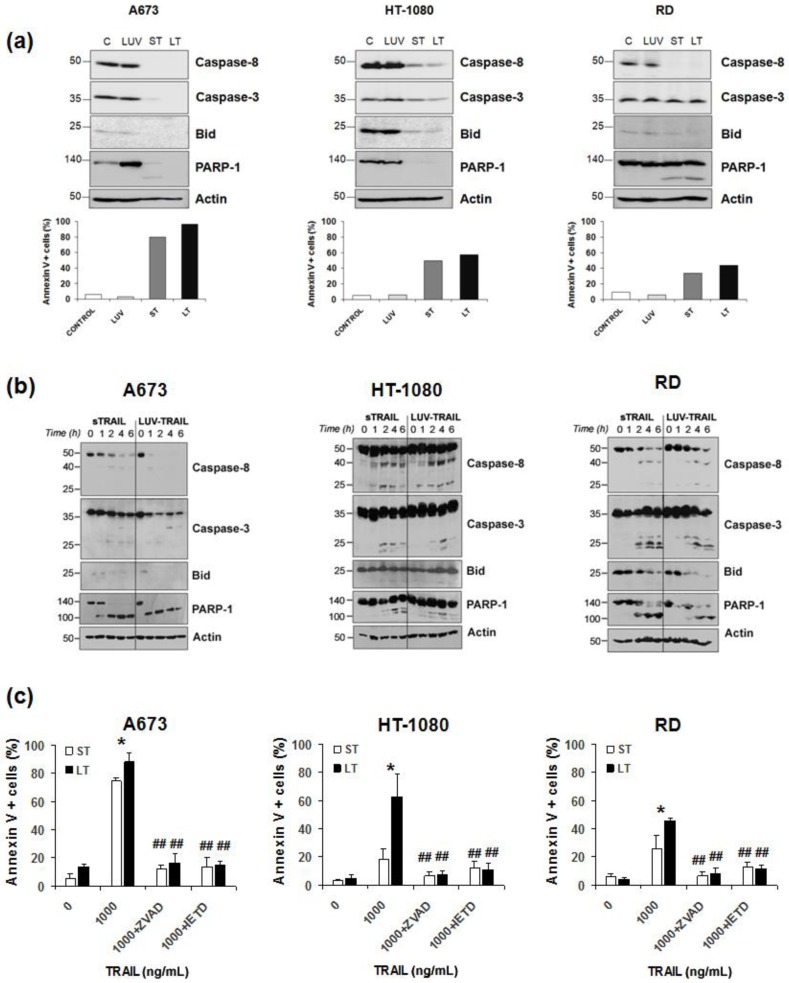
(**a**) Analysis of caspase activation in human sarcoma cells. Cells were untreated (Control, designed as C), or treated with LUVs without TRAIL (LUV), sTRAIL (ST), and LUV-TRAIL (LT) at 1000 ng/mL for 24 h. After that, cells were lysed, and lysates were subjected to SDS-PAGE and to Western blot analysis. Levels of caspase-8, caspase-3, Bid, and PARP-1 were analyzed using specific antibodies. Level of actin levels was used as a control for equal protein loading. Cell death was measured in parallel by flow cytometry after annexin-V staining (bottom graphs); (**b**) Analysis of time-course caspase activation in human sarcoma cells. Cells were treated with sTRAIL or LUV-TRAIL at 1000 ng/mL at the indicated times. After that, cells were lysed, and lysates were subjected to SDS-PAGE and to Western blot analysis. Levels of caspase-8, caspase-3, Bid, and PARP-1 were analyzed using specific antibodies. Level of actin levels was used as a control for equal protein loading. Caspase activation was evidenced by the disappearance of the pro-forms shown in the Western blot; (**c**) Analysis of cell death inhibition by caspase inhibition. Human sarcoma cells were treated with 1000 ng/mL of both sTRAIL (ST) and LUV-TRAIL (LT) for 24 h previously incubated in presence or absence of the pan-caspase inhibitor z-VAD-fmk (30 µM) and of the specific caspase-8 inhibitor IETD-fmk (30 µM). Graphics show the mean ± SD of the cell death of treated cells expressed as percentage of at least three experiments. * *p* < 0.05. (ST versus LT). ## *p* < 0.01 (ST versus ST + caspase inhibitors and, LT versus LT + caspase inhibitors).

Next, the implication of caspases in the cytotoxicity induced by LUV-TRAIL in sarcoma cells was assessed. For that purpose, sarcoma cells were incubated with sTRAIL or LUV-TRAIL and activation of the main caspases involved in the extrinsic apoptotic pathway was analyzed by Western blot. Activation of both caspase-8 and caspase-3 was clearly increased when sarcoma cells were treated with LUV-TRAIL compared to sTRAIL, as evidenced by the disappearance of the pro-forms of both caspases (Figure 2a). Moreover, cleavage of the specific caspase-3 substrate, PARP-1, and the specific caspase-8 substrate, Bid, correlated with the activation of both caspases -3 and -8, respectively, indicating a fully functional activation of the extrinsic apoptotic pathway upon LUV-TRAIL treatment. When time course assays were performed (Figure 2b), caspase activation was faster in A673 cells when they were treated with LUV-TRAIL, although, as seen previously, both formulations of TRAIL present similar cytotoxicity at 24 h. In HT-1080 cells, similar kinetics was observed at shorter times when they were treated both with sTRAIL and LUV-TRAIL. However, as shown in Figure 2a, caspase activation was greater when HT-1080 cells were treated with LUV-TRAIL in comparison with sTRAIL after 24 h of treatment. These data reflect that LUV-TRAIL required longer time of incubation to induce a greater caspase activation and, hence, a greater cytotoxicity than sTRAIL in HT-1080 cells. In case of RD cells, although no obvious differences could be observed in caspase activation after treatment with sTRAIL or LUV-TRAIL, Bid and PARP-1 degradation was faster when cells were treated with LUV-TRAIL. Finally, to fully assess and characterize the role of caspases in LUV-TRAIL induced cell death, cell death-inhibition assays were performed using the general caspase inhibitor z-VAD-fmk (Figure 2c). As expected, caspase inhibition fully abrogated cell death induced not only by sTRAIL but also by LUV-TRAIL. Moreover, when cells were pre-incubated with the specific caspase-8 inhibitor IETD-fmk, cell death induced by LUV-TRAIL was also fully abrogated, proving that cell death was fully dependent on the activation of the canonical extrinsic apoptotic pathway, ruling out any other form of cell death that could be triggered by TRAIL, such as necroptosis.

### 2.3. Combination of LUV-TRAIL with Anti-Cancer Agents Improved its Cytotoxicity against Human Sarcoma Cells

**Figure 3:**
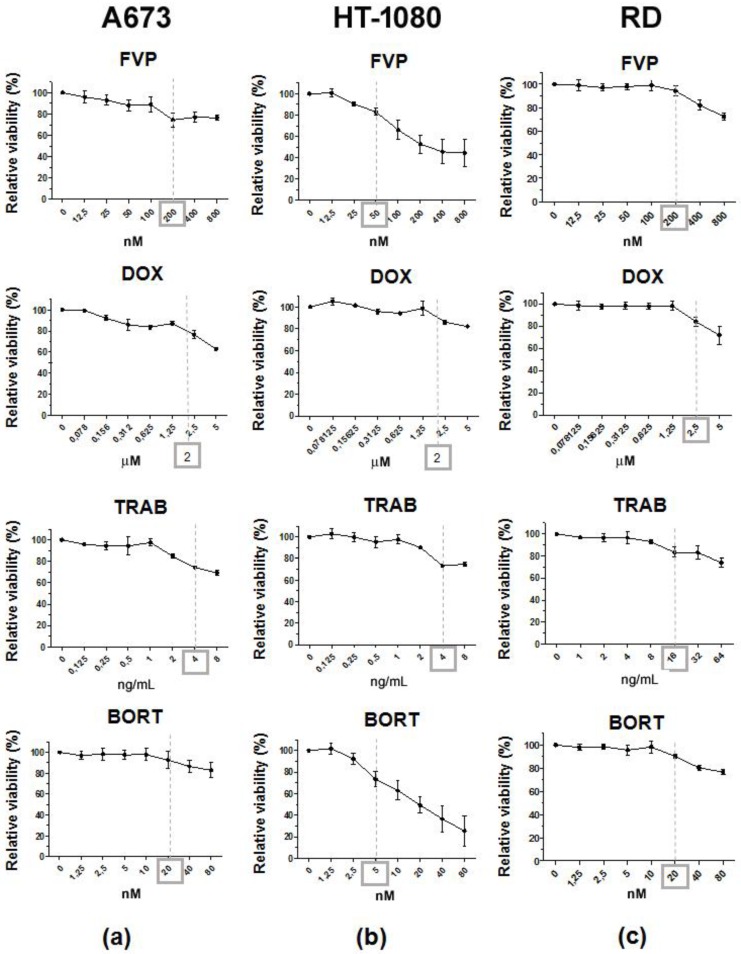
Analysis of the effect of sensitizer agents in cell viability of human sarcoma cells. Dose-response assays using the sensitizer agents: flavopiridol (FVP), doxorubicin (DOX), trabectedin (TRAB), and bortezomib (BORT) were performed on A673 cells (**a**), HT-1080 cells (**b**), and RD cells (**c**). Cell viability was assessed by MTT assay after 24 h. The results were expressed as the mean ± SD of at least three experiments. Marked doses of each drugs were selected for further experiments of sensitization.

Although LUV-TRAIL showed a greater cytotoxic effect than sTRAIL and was able to overcome sTRAIL resistance in HT-1080 and RD cells, LUV-TRAIL was only capable of inducing a decrease of cell viability of around 50% in both sarcoma cell lines at the 1000 ng/mL dose. Therefore, we decided to combine LUV-TRAIL with several anti-cancer agents such as flavopiridol (FVP), doxorubicin (DOX), trabectedin (TRAB), and bortezomib (BORT) in order to improve LUV-TRAIL-induced cytotoxicity. First of all, dose-response assays were carried out to analyze the cytotoxic effect of all anti-cancer agents used alone (Figure 3). Aiming for a sensitizing effect rather than an additive effect, sub-toxic doses of the different drugs were selected by choosing the maximum concentrations not inducing a decrease of cell viability greater than 20% (marker doses for each anti-cancer drug in Figure 3).

After that, dose-response assays were carried out by pre-incubating sarcoma cells with the selected doses of each anti-cancer drug for 2 h before treating with either sTRAIL or LUV-TRAIL.

**Figure 4:**
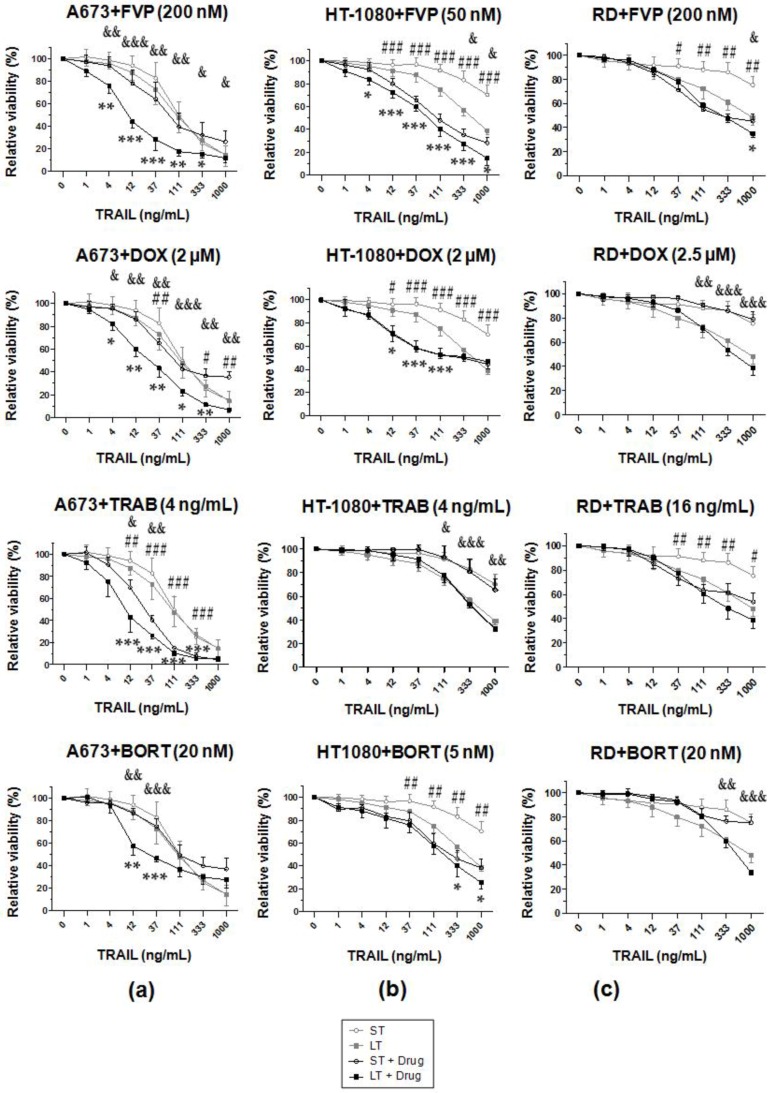
Analysis of cell viability of human sarcoma cell lines after treatment with sTRAIL or LUV-TRAIL in combination with sensitizer agents. Cells were pre-incubated for 2 h with sensitizer drugs: flavopiridol (FVP), doxorubicin (DOX), trabectedin (TRAB), and bortezomib (BORT) at indicated doses. Afterwards, sTRAIL (ST) or LUV-TRAIL (LT) were added at the indicated concentrations and left overnight. The following day, cell viability was measured by the MTT assay. In every graph, the results from treatments with ST and LT in the absence of drugs are superposed in light grey to show the sensitizing effect more clearly. Graphs show the mean ± SD of at least independent experiments. Asterisk signs indicate significance between LT alone with LT in combination with a given drug (* *p* < 0.05, ** *p* < 0.005, *** *p* < 0.001). Pound signs indicate significance between ST alone with ST in combination with a given drug (# *p* < 0.05, ### *p* < 0.005, ### *p* < 0.001). A673 cells (**a**), HT-1080 cells (**b**), and RD cells (**c**). Ampersand signs indicate significance between ST in combination with a given drug with LT in combination with same drug. (& *p* < 0.05, && *p* < 0.005, &&& *p* < 0.001).

In the case of A673 cells, although both sTRAIL and LUV-TRAIL induced a great decrease of cell viability at the highest dose used (1000 ng/mL), all different anti-cancer drugs were able to sensitize to LUV-TRAIL at intermediate doses (Figure 4a).

In HT-1080 cells, although FVP greatly sensitized to both sTRAIL and LUV-TRAIL, the combination with LUV-TRAIL was the most cytotoxic (Figure 4b). Combination of TRAIL with DOX sensitized to both sTRAIL and LUV-TRAIL to the same extent. Similarly, BORT equally sensitized the cells to both sTRAIL and LUV-TRAIL, whereas TRAB did not show any sensitizing effect whatsoever (Figure 4b).

Finally, in RD cells, combination of both sTRAIL and LUV-TRAIL with FVP or TRAB induced similar cytotoxocity levels, whereas DOX and BORT did not sensitize to neither sTRAIL nor LUV-TRAIL (Figure 4c).

### 2.4. Combination of FVP with LUV-TRAIL Induced Apoptotic Cell Death and Decreased Long-Term Clonogenic Survival of Human Sarcoma Cells

**Figure 5:**
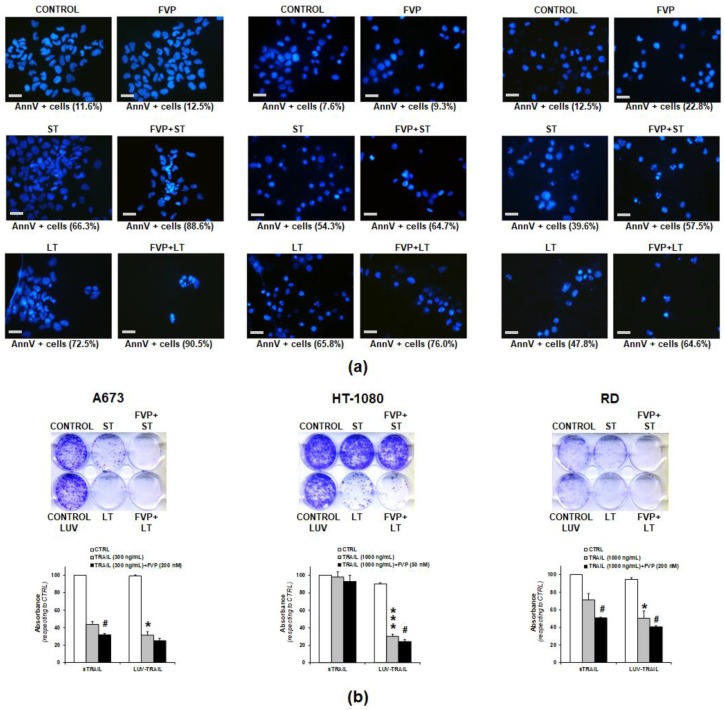
(**a**) Analysis of nuclear morphological changes after combination of LUV-TRAIL with FVP. Human sarcoma cells were pre-incubated for 1 h with flavopiridol (FVP, 200 nM for A673 and RD cells, 50 nM for HT-1080 cells). Afterwards, sTRAIL (ST) or LUV-TRAIL (LT) were added (300 ng/mL for A673 cells and 1000 ng/mL for HT-1080 and RD cells) and left overnight. The following day, nuclear staining was performing by using Hoechst 33342. As control, cells were untreated (CONTROL) or treated only with FVP (FVP) at above indicated doses. Original magnification at 400×. Scale bar = 20 µM. Cell death was measured in parallel by flow cytometry after annexin-V staining (bottom graphs); (**b**) Analysis of clonogenic survival after combination of LUV-TRAIL with FVP. Clonogenic assay was performed to analyze long-term survival in human sarcoma cells after treatment with sTRAIL (ST) or LUV-TRAIL (LT) for 24 h (300 ng/mL for A673 cells and 1000 ng/mL for HT-1080 and RD cells). As indicated, cells were pre-incubated with FVP (FVP, 200 nM for A673 and RD cells, 50 nM for HT-1080 cells) for 1 h. Upper panels show 6-well plates seeded with human sarcoma cells and stained with crystal violet after 11 days. Bottom panels show the quantification of crystal violet absorbance after solubilizing in DMSO and measuring absorbance at 550 nm. Graphic shows the mean ± SD of the absorbance of treated cells expressed as percentage with respect to the untreated cells (control). Asterisk signs indicate significance between LT and LT (* *p* < 0.05, ** *p* < 0.005, *** *p* < 0.001). Pound signs indicate significance between TRAIL (ST or LT) alone with TRAIL in combination with FVP (# *p* < 0.05).

According to the results obtained, combination of LUV-TRAIL with FVP greatly enhanced their cytotoxic ability in all sarcoma cell lines tested. Next, we sought to confirm whether the decrease of cell viability observed in Figure 4 was due to the induction of apoptosis or to a cytostatic effect. First, appearance of typical apoptotic features was assessed by microscopy (Figure 5a). In all cases, cells treated with sTRAIL and LUV-TRAIL alone or in combination with FVP exhibited the typical apoptotic nuclear morphological changes such as blebbing, nuclear fragmentation, and chromatin condensation (Figure 5a). Moreover, these nuclear apoptotic morphological changes were more pronounced when both forms of TRAIL were combined with FVP compared with treatments with TRAIL alone (both sTRAIL and LUV-TRAIL). Finally, apoptotic morphological changes correlated in all cases with induction of apoptosis carried out in parallel using annexin V staining (Figure 5a, showed as percentage of annexin V positive cells below each picture).

Next, we sought to analyze the long-term cytotoxic effect of the combinations of both forms of TRAIL alone or in combination with FVP. For this purpose, clonogenic assays were carried out. (Figure 5b). Notably, liposomes alone (without TRAIL anchored on their surface) were added as controls to rule out any long-term cytotoxic effect of the lipid nanoparticles (see controls for LUV-TRAIL in Figure 5b). Treatment with LUV-TRAIL for 24 h significantly delayed clonogenic survival of sarcoma cells when compared to sTRAIL, indicating that LUV-TRAIL not only showed a greater cytotoxic short-term effect than sTRAIL but also showed a long-term effect affecting to survival and proliferation of sarcoma cells. Remarkably, when both forms of TRAIL were combined with FVP, the clonogenic suvival of sarcoma cells was enhanced compared to TRAIL alone, being again more pronounced in the case of LUV-TRAIL.

### 2.5. Combination of FVP with LUV-TRAIL Activated the Caspase Cascade More Efficiently than with sTRAIL in Human Sarcoma Cells

**Figure 6:**
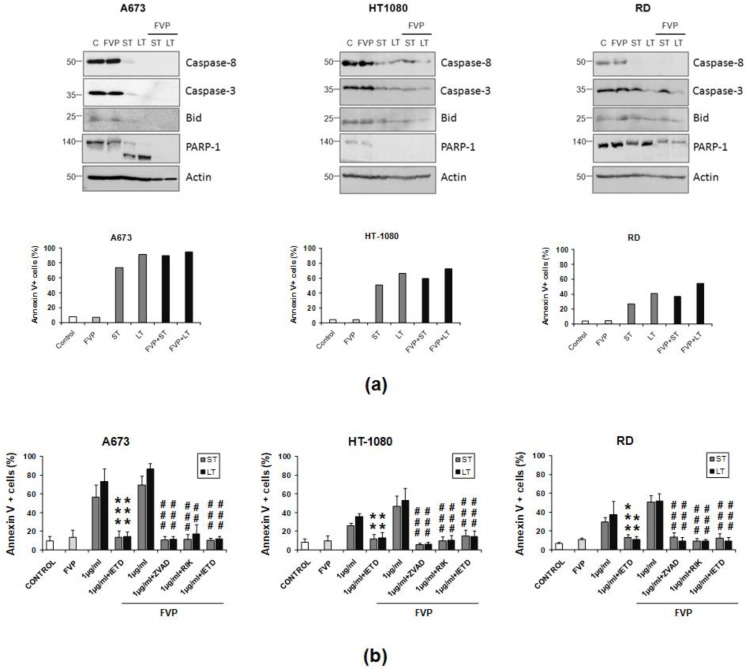
(**a**) Caspase activation induced by combined treatments in human breast tumor cells. Cells were pre-incubated for 1 h with FVP (200 nM for A673 and RD cells, 50 nM for HT-1080 cells) and then treated with indicated doses of sTRAIL (ST) or LUV-TRAIL (LT) overnight. After that, cells were lysed and levels of caspase-8, caspase-3, Bid, and PARP-1 were analyzed by Western blot analysis using specific antibodies. Actin levels were also determined as a control for equal protein loading (upper panels). As control, cells were untreated (C) or treated only with FVP at above indicated doses. An aliquot of untreated and treated human sarcoma cells was collected in parallel and apoptosis was analyzed by annexin V staining using flow cytometry (lower panels); (**b**) Analysis of apoptosis inhibition by caspase inhibition. Human sarcoma cells were treated with indicated doses of sTRAIL (ST) and LUV-TRAIL (LT) previously pre-incubated in presence or absence of FVP (200 nM for A673 and RD cells, 50 nM for HT-1080 cells). Combined treatment was also performed pre-incubating with the TRAIL-blocking antibody RIK2 (500 ng/mL), with the pan-caspase inhibitor z-VAD-fmk (30 µM) and with the specific caspase-8 inhibitor IETD-fmk (30 µM). Graphics show the percentage of annexin-V positive cells analyzed by flow cytometry as the mean ± SD of at least three experiments. ** *p* < 0.01, *** *p* < 0.001 (ST versus ST + RIK and, LT versus LT + RIK). ## *p* < 0.01, ### *p* < 0.001 (ST versus ST + caspase inhibitors and, LT versus LT + caspase inhibitors).

After assessing the greater cytotoxic effect of the combination of LUV-TRAIL with FVP at both short and long term, activation of the main caspases involved in the extrinsic apoptotic pathway (caspase-8 and caspase-3) was analyzed by Western blot. As aforementioned (Figure 2), both sTRAIL and LUV-TRAIL induced the activation of the extrinsic apoptotic pathway, as shown by the clear decrease of the pro-forms of both caspases-8 and -3, as well as of Bid and PARP-1, in the three sarcoma cell lines tested (Figure 6a). Moreover, caspase activation correlated in all cases with induction of apoptosis as indicated by annexin V staining performed in parallel (Figure 6a, lower panels). Pretreatment with FVP greatly increased the disappearance of all proteins, proving that FVP sensitization to both sTRAIL and LUV-TRAIL relies on an enhanced activation of the extrinsic apoptotic pathway. Importantly, FVP did not induce any detectable caspase activation in any sarcoma cell tested (Figure 6a, lower panels).

On the other hand, cell death induced by combination of either sTRAIL or LUV-TRAIL with FVP was fully inhibited by the general caspase inhibitor z-VAD-fmk and the specific caspase inhibitor IETD-fmk. These data demonstrated that cell death induced by the combination of FVP with TRAIL (sTRAIL and LUV-TRAIL) was a caspase-dependent apoptotic process through the activation of the extrinsic apoptotic pathway by caspase-8 (Figure 6b). In this line, cell death induced by the combination of FVP with both formulations of TRAIL was exclusively dependent of TRAIL since blocking TRAIL-signaling with the TRAIL-blocking antibody RIK2 entirely abrogated apoptosis induced by combination of FVP with both sTRAIL and LUV-TRAIL (Figure 6b).

### 2.6. FVP Diminished Expression of Anti-Apoptotic Proteins in Human Sarcoma Cells

**Figure 7:**
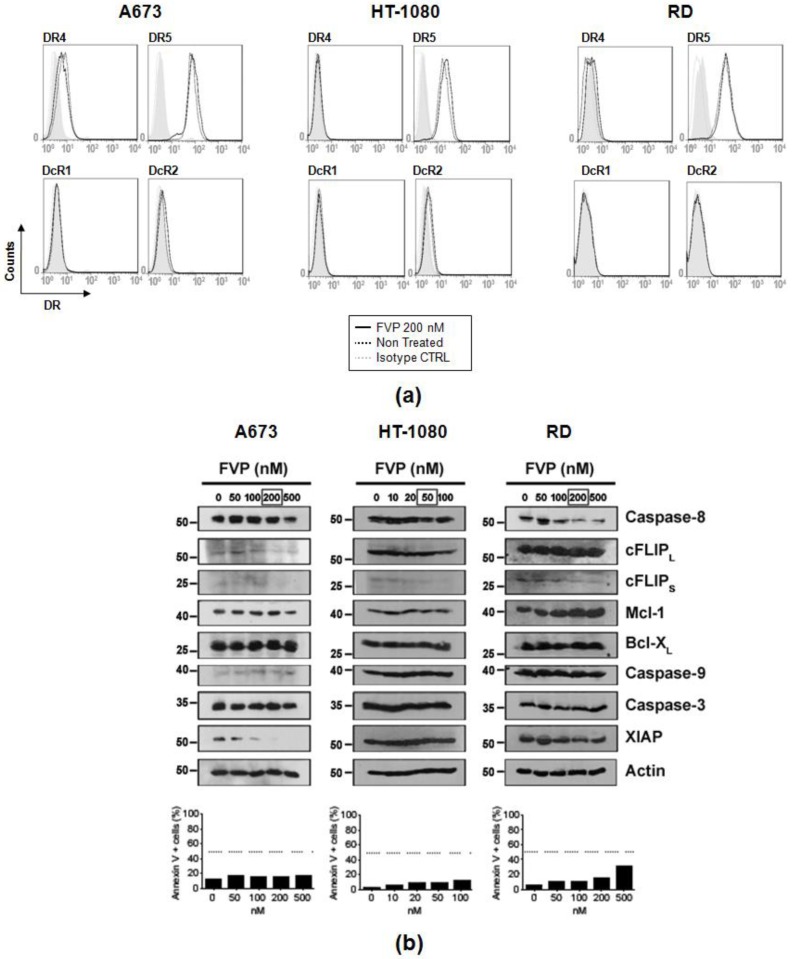
(**a**) Effect of FVP in death receptor (DR) surface expression in human sarcoma cells. Surface expression of DR4, DR5, DcR1, and DcR2 in A673, HT-1080, and RD cells untreated (black dotted line) or treated with 200 nM FVP for 16 h (black line) was analyzed by flow cytometry. Grey dotted line indicates isotype control and grey histogram indicates unlabeled cells. (**b**) Effect of FVP in protein expression in human sarcoma cells. Flavopiridol (FVP) were used at indicated doses was used to treat human sarcoma cell lines for 16 h. After that, cells 10^6^ cells were lysed and expression of caspase-8, FLIP, Mcl-1, Bcl-X_L_, caspase-9, caspase-3, and XIAP were analyzed by Western blot using specific antibodies. Actin levels were also determined as a control for equal protein loading (upper panels). An aliquot of untreated and treated human sarcoma cells was collected in parallel and labeled with annexin-V. Apoptosis was analyzed by flow cytometry (lower panels).

After assessing the enhanced cytotoxic activity showed by combined treatments of both TRAIL formulations with FVP, we analyzed the underlying mechanism of FVP-induced sensitization to TRAIL. Analysis of FVP effect on the surface expression of pro-apoptotic receptors, DR4 and DR5, and decoy receptors, DcR1 and DcR2, showed no significant changes of death receptors (DR) upon treatment with FVP (Figure 7a).

Therefore, we investigated the expression of different proteins involved in the regulation of TRAIL-induced apoptosis in sarcoma cells by Western blot. As previously observed, we confirmed that FVP did not significantly induce apoptosis at the doses used (Figure 7b, bottom panels). Flavopiridol induced a clear decrease of the anti-apoptotic protein FLIP (mainly cFLIP_S_) in all sarcoma cell lines tested (Figure 7b, upper panels). Moreover, in A673 cells a clear decrease of the anti-apoptotic protein XIAP was observed. Finally, expression of other anti-apoptotic proteins such as Mcl-1 and Bcl-X_L_ did not vary after FVP treatment in any sarcoma cell line tested (Figure 7b).

## 3. Discussion

Sarcomas are relatively rare malignant tumors of mesenchymal origin and constitute about 1% of all cancers. Current treatment of sarcomas implies a multidisciplinary approach including surgery, chemotherapy, and radiotherapy [31]. This has led to an improvement in prognosis of patients with sarcoma (overall survival about 50%) [32]. However, the implementation of novel therapies could help to improve the survival of patients suffering from sarcoma in the future. In this line, TRAIL has been tested in distinct types of sarcoma both in pre-clinical studies and clinical trials [5]. However, the potential of TRAIL as possible treatment in sarcomas has been explored in all cases using the soluble form of TRAIL [28,33,34].

Our group previously generated a new TRAIL formulation (LUV-TRAIL) based on tethering human recombinant TRAIL on the surface of artificial LUV-type liposomes. The improved bioactivity of LUV-TRAIL has been validated in a broad panel of human cancer cells derived from hematological malignancies [19,20,21], as well as epithelial cancer cells both in vitro and in vivo [22,23,24]. Notably, although LUV-TRAIL was more cytotoxic than sTRAIL against cancer cells, it lacked toxicity against normal cells both in vitro [19] and in vivo [23]. The enhanced bioactivity of LUV-TRAIL compared with sTRAIL relied on its capability of forming supra-trimeric populations of high molecular order which were not present in sTRAIL [24]. These supra-trimeric populations of high molecular order present in LUV-TRAIL formulation promoted the clear formation of DR5 oligomers on the target cells. In short, liposome-bound TRAIL induced superior DR5 clustering, enhancing DISC recruitment and, consequently, triggering caspase activation more efficiently than the sTRAIL [20,24].

In the present work, we have extended the study of the anti-tumor potential of LUV-TRAIL to sarcomas. LUV-TRAIL was capable of inducing cell death more efficiently than sTRAIL both in TRAIL-sensitive sarcoma cells (A673) cells and TRAIL-resistant sarcoma cells (HT-1080 and RD). Cell death induced by LUV-TRAIL in sarcoma cells was specifically due to TRAIL since pre-incubation with the neutralizing antibody RIK2 before treatment fully abrogated cell death induced by LUV-TRAIL. In this line, LUVs without TRAIL anchoring on their surface did not show any cytotoxicity in sarcoma cells, confirming that LUV-TRAIL-induced cell death was in fact fully attributable to TRAIL. Moreover, cell death induced by LUV-TRAIL was a caspase-dependent apoptotic process through the activation of the extrinsic apoptotic pathway by caspase-8 as evidenced by the fact that the pan-caspase inhibitor zVAD-fmk and the specific caspase-8 inhibitor IETD-fmk completely inhibited LUV-TRAIL-induced cell death.

It is interesting that in the several studies that have explored the anti-tumor potential of TRAIL in sarcomas, this death ligand has been used in combination with other drugs, indicating that some sarcoma types are partially resistant to the sTRAIL [27,28,33,35,36,37]. In this line, although LUV-TRAIL showed more pro-apoptotic potential than sTRAIL in sarcoma cell lines, they only induced a moderate cytotoxic effect in sTRAIL-resistant HT-1080 and RD cells. Therefore, we decided to combine LUV-TRAIL with several drugs described to sensitize sarcoma cells to TRAIL-induced apoptosis such as doxorubicin (DOX) [27], trabectedin (TRAB) [28], bortezomib (BORT) [38], and flavopiridol (FVP). Previous studies of our group on a breast cancer model, sensitization experiments also using flavopiridol were performed pre-incubating cancer cells before treatment with TRAIL (sTRAIL or LUV-TRAIL), as well as simultaneous treatment with FVP and both forms of TRAIL [23]. On that model, no differences were observed when cells were previously treated with FVP and then with TRAIL in comparison with simultaneous treatment. In this line, other studies carried out by us using multiple myeloma cells do not show differences between pre-incubation strategy and simultaneous treatment when TRAIL (sTRAIL or LUV-TRAIL) is combined with drugs (data not shown).

Among them, FVP was proven as the most efficient in the three sarcoma cell lines tested. Flavopiridol is a semisynthetic flavone that has showed a potent inhibitory effect on cell proliferation in sarcoma cells [39,40,41]. Flavopiridol has also been used in clinical trials, and even though was not effective as monotherapy [42], FVP potentiated the anti-tumor activity of other anti-tumor agents when was used in combination [43,44].

Our results demonstrate that FVP greatly sensitized not only to sTRAIL but also, and largely, to LUV-TRAIL. The cytotoxic effect of LUV-TRAIL in combination with FVP was fully attributed to TRAIL since the selected doses of FVP did not induce cell death per se. In this line, pre-incubation with the neutralizing antibody RIK2 fully inhibited cell death induced by the combined treatment. Cell death induced by the combination of FVP with TRAIL showed the typical morphological changes of apoptotic cell death such as blebbing, nuclear fragmentation, and chromatin condensation. Furthermore, this sensitization resulted in an increased activation of both caspase-8 and -3, which was completely inhibited by the pan-caspase inhibitor z-VAD-fmk and also by the caspase-8 inhibitor IETD-fmk. Altogether, these data indicate that combination of FVP with LUV-TRAIL was a caspase-dependent apoptotic process through the activation of the extrinsic apoptotic pathway. Moreover, when long-term effect of LUV-TRAIL was analyzed, this novel TRAIL formulation inhibited clonogenic cell growth largely than sTRAIL. Moreover, combination of FVP with both forms of TRAIL, mainly with LUV-TRAIL, enhanced long-term effect indicating that the combined treatment not only was more effective at short-term but also was prolonged over time as shown in clonogenic assays.

Several mechanisms have been described to explain FVP-induced TRAIL-sensitization, among them up-regulation of DR expression [23] or inactivation of anti-apoptotic proteins such as FLIP, XIAP, Mcl-1, or survivin [25,45,46]. Trying to ascertain the mechanisms involved in the synergy observed between FVP and LUV-TRAIL, we firstly analyzed DR expression upon FVP treatment. We previously demonstrated that DR up-regulation was a decisive sensitizing mechanism of FVP to TRAIL-induced apoptosis in breast cancer cells [23]. However, FVP did not modify either the expression of the pro-apoptotic TRAIL receptors or that of the decoy receptors in sarcoma cell lines, underscoring that sensitization mechanisms are different depending on the cancer type studied. In this line, we also did not observe DR up-regulation in lung cancer cells upon FVP treatment [22]. On the other hand, it has been described that FVP promotes degradation of anti-apoptotic proteins such as cFLIP, Mcl-1, and XIAP [25,45,46]. In agreement with previous studies, we observed that FVP induced a clear decrease of FLIP in all sarcoma cell lines tested. In fact, FVP induced a clear down-regulation of the short isoform of cFLIP (cFLIP_S_), while no changes were observed for the long isoform of cFLIP (cFLIP_L_), with the exception of A673 cells. It is noteworthy to point out that cFLIP_S_ is considered to be solely an anti-apoptotic protein promoting caspase-8 inhibition, while the role of cFLIP_L_ is not clear, and seems that the long isoform of cFLIP promotes or inhibits apoptosis depending on the relative amounts of both caspase-8 and cFLIP_L_ [47,48]. Interestingly, FVP also induced a clear decrease of XIAP expression in A463 cells, which also resulted to be the most sensitive cell line to the combined treatment of FVP with both TRAIL formulations. Although we have not performed specific experiments to address the precise contribution of XIAP down-regulation to FVP-mediated sensitization to TRAIL, this could at least partially explain the higher sensitizing effect of FVP in these cells compared to the other cell lines.

Summarizing, this study shows that LUV-TRAIL significantly improves the bioactivity of sTRAIL on sarcoma cells. Furthermore, the combination of LUV-TRAIL with FVP increased even more the cytotoxic potential of LUV-TRAIL, opening the door to new TRAIL-sensitization strategies of which LUV-TRAIL mainly could benefit. In conclusion, the present study validates our novel formulation of TRAIL based on anchoring this death ligand on liposome surface in sarcoma and could be of relevance in a future clinical application of TRAIL in this type of cancer.

## 4. Materials and Methods

### 4.1. Preparation of Lipid Nanoparticles Decorated with Soluble Recombinant TRAIL

LUV (Large Unilamellar Vesicles)-type lipid nanoparticles with soluble recombinant TRAIL (sTRAIL) tethered on their surface was performed as previously described [19,49]. Briefly, a mixture of phosphatidylcholine (PC), sphingomyelin (SM), cholesterol (CHOL), and 1,2-dioleoyl-sn-glycero-3-{[*N*-(5-amino-1-carboxypentyl)-iminodiacetic acid]succinyl} (nickel salt) (DOGS-NTA-Ni) (all from Avanti Polar Lipids, Alabaster, AL; USA) in the weight ratio of 55:30:10:5 were firstly dried under a nitrogen and next under vacuum. Lipid mixture with a composition resembling that of natural exosomes was resuspended in KHE buffer (100 mM KCl, 10 mM HEPES, pH 7.0, containing 0.1 m MEDTA). After that, resuspended lipids were freeze-thawed 10 times and extruded 10 times through two polycarbonate membranes with a pore size of 0.2 µm (Whatman, Maidstone, UK) using an extruder (Northern Lipids, Burnaby, BC, Canada). LUV were incubated in KHE buffer for 30 min at 37 °C with soluble recombinant TRAIL (sTRAIL), corresponding to amino acids 95–281 with a 6-histidine tag in its N-terminal extreme cloned into the pET-28c plasmid (Novagen, kindly provided by Dr. Marion MacFarlane) [50]. Then LUV with sTRAIL tethered on their surface (LUV-TRAIL) were ultracentrifugated for 6 h at 100,000 revolutions per minute at 4 °C, supernatant was removed, and finally, the pellet containing LUV-TRAIL was resuspended in an equal volume of sterile KHE buffer.

### 4.2. Cell Culture and Cytotoxicity Assays

A673 cells (derived from Ewing’s sarcoma), HT-1080 cells (derived from fibrosarcoma), and RD cells (derived from rhabdomyosarcoma) were obtained from American Type Culture Colection (ATCC, Manassas, VA, USA). Sarcoma cell lines were routinely cultured in Dulbecco’s Modified Eagle Medium (DMEM) medium supplemented with 10% fetal bovine serum (FBS), 2 mM l-glutamine and penicillin/streptomycin (i.e., complete medium) at 37 °C with a 5% CO_2_.

### 4.3. Cell Viability Assays

For cell viability quantification, cells (2.5 × 10^4^ cells/well) were seeded in 96-well plates (100 μL/well) in complete medium and left overnight to be attached to the bottom. Cells were then treated with different concentrations (1–1000 ng/mL) of sTRAIL or LUV-TRAIL for 24 h. Cell viability was evaluated by a modification of 3-[4,5-di-methylthiazol-2-yl]-2,5-diphenyltetrazolium bromide (MTT) method of Mosmann as previously described [51]. Data was expressed as the percentage of cell viability with respect to control cells (untreated cells for sTRAIL, and cells treated with LUVs without TRAIL for LUV-TRAIL).

### 4.4. Cytotoxicity Assays

Cytotoxicity assays were performed as follows: 2 × 10^4^ cells were seeded in 96-well plates in complete medium and left overnight to be attached to the bottom. After that, cells were treated with different concentrations of sTRAIL or LUV-TRAIL (1–1000 ng/mL) for 24 h. Then, apoptosis was measured by analyzing phosphatidyl-serine exposure on cell surface was analyzed to quantify apoptosis. For that, cells were incubated with 0.5 µg/mL annexin-V-APC, Immunostep, Salamanca, Spain) in annexin-binding buffer (ABB, 140 mM NaCl, 2.5 mM CaCl_2_, 10 mM HEPES/NaOH, pH 7.4) for 15 mins at room temperature. Finally, apoptosis quantification was carried out using a FACSCalibur flow cytometer and CellQuest software (BD Biosciences, Franklin Lakes, NY, USA).

Cell death inhibition assays were carried out by using the blocking anti-human TRAIL mAb (500 ng/mL, clone RIK2, BD Biosciences), with the pan-caspase inhibitor z-VAD-fmk (30 µM, Bachem, Bubendorf, Switzerland) or with the specific caspase-8 inhibitor IETD-fmk (30 µM, Bachem). Cells were pre-incubated with RIK2, z-VAD-fmk, or IET-fmk for 1 h prior to the addition of TRAIL (both sTRAIL and LUV-TRAIL).

### 4.5. Clonogenic Assay

Clonogenic survival was analyzed as previously described [22,23,24]. Briefly, 5000 cells per well were seeded into 6-well plates and left to be attached to the bottom overnight. The following day, cells were incubated with sTRAIL or LUV-TRAIL (300 ng/mL for A673 cells and 1000 ng/mL for HT-1080 and RD cells) for 24 h in presence or absence of FVP (50 nM for HT-1080 cells and 200 nM for A673 and RD cells). Then, culture medium was removed and replaced with fresh medium, and surviving cells were cultured for 10 days. After that, cells were washed twice with PBS, fixed with pure methanol for 30 min at 4 °C, and stained with crystal violet (1% in 50% ethanol). The measurement of the absorbance at 550 nm after dissolving crystal violet with DMSO was performed for quantifying the clonogenic assays. Data were expressed as the percentage of absorbance with respect to the respective control (untreated cells).

### 4.6. Western Blot Analysis

The study of the expression of the main proteins involved in the extrinsic apoptotic pathway was carried out using Western blot analysis as previously described [19,20,21]. Briefly, cells (5 × 10^6^) were lysed at 4 °C with 100 µL of a buffer containing 1% Triton X-100 and protease and phosphatase inhibitors. Then, lysated cells were separated by 12% SDS-PAGE, transferred to PVDF membranes, and blocked with TBS-T buffer (10 mM Tris/HCl, pH 8.0, 0.12 M NaCl, 0.1% Tween-20, 0.05% sodium azide) containing 5% skimmed milk. PVDF membranes were incubated with mAbs against caspase-8 (BD Biosciences), caspase-3 (Cell Signaling, Danvers, MA; USA), Bid (BD Biosciences), PARP-1 (BD biosciences), caspase-9 (MBL, Woburn, MA; USA), cFLIP (clone NF6, Enzo, Farmingdale, NY, USA), Mcl-1 (Santa Cruz Biotech, Dallas, TX), Bcl-xL (Cell Signaling), or XIAP (BD Biosciences) in TBS-T containing 2% skimmed milk. Anti-β-actin mAb (Sigma, Saint Louis, MO; USA) was used as protein loading control. Pierce ECL Western Blotting Substrate (when used horseradish peroxidase-labeled secondary antibody, Life Technologies, Carlsbad, CA, USA) or the CDP-Star substrate (when used phosphatase alkaline-labeled secondary antibody, Merck, Darmstadt, Germany) were used to display the proteins.

### 4.7. Surface Expression of Death Receptors

The analysis of surface expression of death receptors was performed as follows: 1 × 10^5^ cells were incubated with either anti-DR4, anti-DR5, anti-DcR1, anti-DcR2 monoclonal antibodies or isotype control, all of them PE-conjugated (eBioscience, San Diego, CA, USA) in PBS containing 5% FCS for 30 min at room temperature. Then, cells were analyzed by using a FACSCalibur flow cytometer and Cell Quest software (BD Biosciences).

### 4.8. Nuclear Staining

Hoechst 33342 staining was carried out for analyzing the nuclear morphological changes. Briefly, cells were seeded (10^5^ cells) in 24-well plates in complete medium and incubated in presence or absence of FVP for 1 h (200 nM for A673 and RD cells and 50 nM for HT-1080 cells). After that, cells were treated with sTRAIL or LUV-TRAIL (1000 ng/mL) overnight. Then, cells were fixed with 4% PFA for 30 min, washed with PBS, and labeled with 1 µg/mL of Hoechst 33342 (Invitrogen, Dublin, Ireland). Finally, photographs were taken using a fluorescence microscope (E600/E400, Nikon, Tokio, Japan) equipped with digital photograph system (DXM 1200F, Nikon) at original magnification at 400×.

### 4.9. Statistical Analysis

GraphPad Prism 5 software was used to carry out computer-based statistical analysis. Results showed indicate the mean ± SD of at least three different experiments. Student’s t test for non-paired variants was performed to evaluate statistical significance. A *p* < 0.05 value was considered to be significant.

## References

[1] Siegel R.L., Miller K.D., Jemal A. (2018). Cancer statistics, 2018. CA Cancer J. Clin..

[2] Borden E.C., Baker L.H., Bell R.S., Bramwell V., Demetri G.D., Eisenberg B.L., Fletcher C.D., Fletcher J.A., Ladanyi M., Meltzer P. (2003). Soft tissue sarcomas of adults: State of the translational science. Clin. Cancer Res..

[3] Wilky B.A., Jones R.L., Keedy V.L. (2017). The Current Landscape of Early Drug Development for Patients with Sarcoma. Am. Soc. Clin. Oncol. Educ. Book.

[4] Kawai A., Yonemori K., Takahashi S., Araki N., Ueda T. (2017). Systemic Therapy for Soft Tissue Sarcoma: Proposals for the Optimal Use of Pazopanib, Trabectedin, and Eribulin. Adv. Ther..

[5] Gamie Z., Kapriniotis K., Papanikolaou D., Haagensen E., Da Conceicao Ribeiro R., Dalgarno K., Krippner-Heidenreich A., Gerrand C., Tsiridis E., Rankin K.S. (2017). TNF-related apoptosis-inducing ligand (TRAIL) for bone sarcoma treatment: Pre-clinical and clinical data. Cancer Lett..

[6] Pitti R.M., Marsters S.A., Ruppert S., Donahue C.J., Moore A., Ashkenazi A. (1996). Induction of apoptosis by Apo-2 ligand, a new member of the tumor necrosis factor cytokine family. J. Biol. Chem..

[7] Wiley S.R., Schooley K., Smolak P.J., Din W.S., Huang C.P., Nicholl J.K., Sutherland G.R., Smith T.D., Rauch C., Smith C.A. (1995). Identification and characterization of a new member of the TNF family that induces apoptosis. Immunity.

[8] Holland P.M. (2014). Death receptor agonist therapies for cancer, which is the right TRAIL?. Cytokine Growth Factor Rev..

[9] Lemke J., von Karstedt S., Zinngrebe J., Walczak H. (2014). Getting TRAIL back on track for cancer therapy. Cell Death Differ..

[10] Micheau O., Shirley S., Dufour F. (2013). Death receptors as targets in cancer. Br. J. Pharmacol..

[11] Von Karstedt S., Montinaro A., Walczak H. (2017). Exploring the TRAILs less travelled: TRAIL in cancer biology and therapy. Nat. Rev. Cancer.

[12] Martinez-Lostao L., Marzo I., Anel A., Naval J. (2012). Targeting the Apo2L/TRAIL system for the therapy of autoimmune diseases and cancer. Biochem. Pharmacol..

[13] Wajant H., Gerspach J., Pfizenmaier K. (2013). Engineering death receptor ligands for cancer therapy. Cancer Lett..

[14] De Miguel D., Lemke J., Anel A., Walczak H., Martinez-Lostao L. (2016). Onto better TRAILs for cancer treatment. Cell Death Differ..

[15] Bernardi S., Secchiero P., Zauli G. (2012). State of art and recent developments of anti-cancer strategies based on TRAIL. Recent Pat. Anticancer Drug Discov..

[16] Lim B., Allen J.E., Prabhu V.V., Talekar M.K., Finnberg N.K., El-Deiry W.S. (2015). Targeting TRAIL in the treatment of cancer: New developments. Expert Opin. Ther. Targets.

[17] Martinez-Lorenzo M.J., Anel A., Gamen S., Monlen I., Lasierra P., Larrad L., Pineiro A., Alava M.A., Naval J. (1999). Activated human T cells release bioactive Fas ligand and APO2 ligand in microvesicles. J. Immunol..

[18] Monleon I., Martinez-Lorenzo M.J., Monteagudo L., Lasierra P., Taules M., Iturralde M., Pineiro A., Larrad L., Alava M.A., Naval J. (2001). Differential secretion of Fas ligand- or APO2 ligand/TNF-related apoptosis-inducing ligand-carrying microvesicles during activation-induced death of human T cells. J. Immunol..

[19] De Miguel D., Basanez G., Sanchez D., Malo P.G., Marzo I., Larrad L., Naval J., Pardo J., Anel A., Martinez-Lostao L. (2013). Liposomes decorated with Apo2L/TRAIL overcome chemoresistance of human hematologic tumor cells. Mol. Pharm..

[20] De Miguel D., Gallego-Lleyda A., Anel A., Martinez-Lostao L. (2015). Liposome-bound TRAIL induces superior DR5 clustering and enhanced DISC recruitment in histiocytic lymphoma U937 cells. Leuk. Res..

[21] De Miguel D., Gallego-Lleyda A., Galan-Malo P., Rodriguez-Vigil C., Marzo I., Anel A., Martinez-Lostao L. (2015). Immunotherapy with liposome-bound TRAIL overcome partial protection to soluble TRAIL-induced apoptosis offered by down-regulation of Bim in leukemic cells. Clin. Transl. Oncol..

[22] De Miguel D., Gallego-Lleyda A., Ayuso J.M., Erviti-Ardanaz S., Pazo-Cid R., del Agua C., Fernandez L.J., Ochoa I., Anel A., Martinez-Lostao L. (2016). TRAIL-coated lipid-nanoparticles overcome resistance to soluble recombinant TRAIL in non-small cell lung cancer cells. Nanotechnology.

[23] De Miguel D., Gallego-Lleyda A., Ayuso J.M., Pawlak A., Conde B., Ochoa I., Fernandez L.J., Anel A., Martinez-Lostao L. (2016). Improved Anti-Tumor Activity of Novel Highly Bioactive Liposome-Bound TRAIL in Breast Cancer Cells. Recent Pat. Anticancer Drug Discov..

[24] De Miguel D., Gallego-Lleyda A., Ayuso J.M., Pejenaute-Ochoa D., Jarauta V., Marzo I., Fernandez L.J., Ochoa I., Conde B., Anel A. (2016). High-order TRAIL oligomer formation in TRAIL-coated lipid nanoparticles enhances DR5 cross-linking and increases antitumour effect against colon cancer. Cancer Lett..

[25] Fandy T.E., Ross D.D., Gore S.D., Srivastava R.K. (2007). Flavopiridol synergizes TRAIL cytotoxicity by downregulation of FLIPL. Cancer Chemother. Pharmacol..

[26] Gamen S., Anel A., Pérez-Galán P., Lasierra P., Johnson D., Piñeiro A., Naval J. (2000). Doxorubicin treatment activates a Z-VAD-sensitive caspase, which causes deltapsim loss, caspase-9 activity, and apoptosis in Jurkat cells. Exp. Cell Res..

[27] Wang S., Ren W., Liu J., Lahat G., Torres K., Lopez G., Lazar A.J., Hayes-Jordan A., Liu K., Bankson J. (2010). TRAIL and doxorubicin combination induces proapoptotic and antiangiogenic effects in soft tissue sarcoma in vivo. Clin. Cancer Res..

[28] Harati K., Chromik A.M., Bulut D., Goertz O., Hahn S., Hirsch T., Klein-Hitpass L., Lehnhardt M., Uhl W., Daigeler A. (2012). TRAIL and taurolidine enhance the anticancer activity of doxorubicin, trabectedin and mafosfamide in HT1080 human fibrosarcoma cells. Anticancer Res..

[29] Balsas P., Lopez-Royuela N., Galan-Malo P., Anel A., Marzo I., Naval J. (2009). Cooperation between Apo2L/TRAIL and bortezomib in multiple myeloma apoptosis. Biochem. Pharmacol..

[30] Shanker A., Brooks A.D., Tristan C.A., Wine J.W., Elliott P.J., Yagita H., Takeda K., Smyth M.J., Murphy W.J., Sayers T.J. (2008). Treating metastatic solid tumors with bortezomib and a tumor necrosis factor-related apoptosis-inducing ligand receptor agonist antibody. J. Natl. Cancer Inst..

[31] Frezza A.M., Stacchiotti S., Gronchi A. (2017). Systemic treatment in advanced soft tissue sarcoma: What is standard, what is new?. BMC Med..

[32] Lazar A.J., Trent J.C., Lev D. (2007). Sarcoma molecular testing: Diagnosis and prognosis. Curr. Oncol. Rep..

[33] Harati K., Emmelmann S., Behr B., Goertz O., Hirsch T., Kapalschinski N., Kolbenschlag J., Stricker I., Tannapfel A., Lehnhardt M. (2016). Evaluation of the safety and efficacy of TRAIL and taurolidine use on human fibrosarcoma xenografts in vivo. Oncol. Lett..

[34] Kang Z., Sun S.Y., Cao L. (2012). Activating Death Receptor DR5 as a Therapeutic Strategy for Rhabdomyosarcoma. ISRN Oncol..

[35] Hotta T., Suzuki H., Nagai S., Yamamoto K., Imakiire A., Takada E., Itoh M., Mizuguchi J. (2003). Chemotherapeutic agents sensitize sarcoma cell lines to tumor necrosis factor-related apoptosis-inducing ligand-induced caspase-8 activation, apoptosis and loss of mitochondrial membrane potential. J. Orthop. Res..

[36] Karlisch C., Harati K., Chromik A.M., Bulut D., Klein-Hitpass L., Goertz O., Hirsch T., Lehnhardt M., Uhl W., Daigeler A. (2013). Effects of TRAIL and taurolidine on apoptosis and proliferation in human rhabdomyosarcoma, leiomyosarcoma and epithelioid cell sarcoma. Int. J. Oncol..

[37] Li X., Huang T., Jiang G., Gong W., Qian H., Zou C. (2013). Proteasome inhibitor MG132 enhances TRAIL-induced apoptosis and inhibits invasion of human osteosarcoma OS732 cells. Biochem. Biophys. Res. Commun..

[38] Lu G., Punj V., Chaudhary P.M. (2008). Proteasome inhibitor Bortezomib induces cell cycle arrest and apoptosis in cell lines derived from Ewing’s sarcoma family of tumors and synergizes with TRAIL. Cancer Biol. Ther..

[39] Cai D., Latham V.M., Zhang X., Shapiro G.I. (2006). Combined depletion of cell cycle and transcriptional cyclin-dependent kinase activities induces apoptosis in cancer cells. Cancer Res..

[40] Jiang J., Matranga C.B., Cai D., Latham V.M., Zhang X., Lowell A.M., Martelli F., Shapiro G.I. (2003). Flavopiridol-induced apoptosis during S phase requires E2F-1 and inhibition of cyclin A-dependent kinase activity. Cancer Res..

[41] Li Y., Tanaka K., Li X., Okada T., Nakamura T., Takasaki M., Yamamoto S., Oda Y., Tsuneyoshi M., Iwamoto Y. (2007). Cyclin-dependent kinase inhibitor, flavopiridol, induces apoptosis and inhibits tumor growth in drug-resistant osteosarcoma and Ewing’s family tumor cells. Int. J. Cancer.

[42] Morris D.G., Bramwell V.H., Turcotte R., Figueredo A.T., Blackstein M.E., Verma S., Matthews S., Eisenhauer E.A. (2006). A Phase II Study of Flavopiridol in Patients with Previously Untreated Advanced Soft Tissue Sarcoma. Sarcoma.

[43] Dickson M.A., Rathkopf D.E., Carvajal R.D., Grant S., Roberts J.D., Reid J.M., Ames M.M., McGovern R.M., Lefkowitz R.A., Gonen M. (2011). A phase I pharmacokinetic study of pulse-dose vorinostat with flavopiridol in solid tumors. Investig. New Drugs.

[44] Luke J.J., D’Adamo D.R., Dickson M.A., Keohan M.L., Carvajal R.D., Maki R.G., de Stanchina E., Musi E., Singer S., Schwartz G.K. (2012). The cyclin-dependent kinase inhibitor flavopiridol potentiates doxorubicin efficacy in advanced sarcomas: Preclinical investigations and results of a phase I dose-escalation clinical trial. Clin. Cancer Res..

[45] Palacios C., Yerbes R., Lopez-Rivas A. (2006). Flavopiridol induces cellular FLICE-inhibitory protein degradation by the proteasome and promotes TRAIL-induced early signaling and apoptosis in breast tumor cells. Cancer Res..

[46] Miyashita K., Shiraki K., Fuke H., Inoue T., Yamanaka Y., Yamaguchi Y., Yamamoto N., Ito K., Sugimoto K., Nakano T. (2006). The cyclin-dependent kinase inhibitor flavopiridol sensitizes human hepatocellular carcinoma cells to TRAIL-induced apoptosis. Int. J. Mol. Med..

[47] Feoktistova M., Geserick P., Kellert B., Dimitrova D.P., Langlais C., Hupe M., Cain K., MacFarlane M., Hacker G., Leverkus M. (2011). cIAPs block Ripoptosome formation, a RIP1/caspase-8 containing intracellular cell death complex differentially regulated by cFLIP isoforms. Mol. Cell.

[48] Pop C., Oberst A., Drag M., Van Raam B.J., Riedl S.J., Green D.R., Salvesen G.S. (2011). FLIP(L) induces caspase 8 activity in the absence of interdomain caspase 8 cleavage and alters substrate specificity. Biochem. J..

[49] Martinez-Lostao L., Garcia-Alvarez F., Basanez G., Alegre-Aguaron E., Desportes P., Larrad L., Naval J., Jose Martinez-Lorenzo M., Anel A. (2010). Liposome-bound APO2L/TRAIL is an effective treatment in a rheumatoid arthritis model. Arthritis Rheum..

[50] MacFarlane M., Ahmad M., Srinivasula S.M., Fernandes-Alnemri T., Cohen G.M., Alnemri E.S. (1997). Identification and molecular cloning of two novel receptors for the cytotoxic ligand TRAIL. J. Biol. Chem..

[51] Mosmann T. (1983). Rapid colorimetric assay for cellular growth and survival: Application to proliferation and cytotoxicity assays. J. Immunol. Methods.

